# Superficial Siderosis and Anticoagulation Therapy: Different Presentations, Different Outcomes

**DOI:** 10.1155/2012/745430

**Published:** 2012-10-09

**Authors:** Rui Duarte Barreto, Luís Ruano, Vítor Tedim Cruz, Carlos Veira, Paula Coutinho

**Affiliations:** Department of Neurology, Entre Douro e Vouga Hospital Center, Rua Dr. Cândido de Pinho, Santa Maria da Feira, 4520-211 Aveiro, Portugal

## Abstract

Superficial siderosis is a potentially manageable neurodegenerative disorder, caused by chronic subarachnoid haemorrhage and iron deposition along the central nervous system surfaces. Association with oral anticoagulant therapy is well known, but its definite role as a causative agent is yet to be clarified. Two Caucasian women, both under long-term oral anticoagulation: a 74 year old woman with slowly progressive hearing loss and mild cerebellar ataxia; a 72 year old woman suffering from behavioural changes, rapidly progressive cognitive decline and latter developing paraparesis. Magnetic resonance imaging showed striking hypointensities along the surfaces of cerebellum, brainstem, frontotemporal cortices, spinal cord, and lumbar arachnoid therefore suggesting superficial siderosis. No specific bleeding source was found in any of the patients. Anticoagulation could not be stopped in the first patient due to a mechanic valve and slowly progressive worsening occurred. In contrast, for the second patient anticoagulation withdrawal was feasible and marked motor and cognitive improvement ensued. Superficial siderosis is associated with unvarying progression, mostly when no direct source of bleeding is identified. Nonetheless, we verified striking motor and cognitive improvement after anticoagulants withdrawal in one of the patients. This may reinforce the need to consider such modifiable factor in future patient management.

## 1. Introduction

Superficial siderosis (SS) is a neurodegenerative disease caused by the deposition of iron metabolites along subpial localizations in the central nervous system (CNS) [1]. Evidence suggests that it results from the release of blood (and iron) in CSF from chronic or recurrent bleeding sources within or adjacent to the CNS. The increase in iron levels leads to the neuroprotective synthesis of hemosiderin and ferritin (less toxic than unbound iron) by microglia cells. When the capacity for local metabolism and clearance is exceeded, iron and related substances accumulate and settle throughout the CNS and meningeal surfaces, with propensity towards the olfactory nerve (I), vestibulocochlear (VII) nerve, cerebellum and spinal anterior horns [2].

The vulnerability of the eighth cranial nerves seems to reside in the long glial segment and passage through the pontine cistern. In the case of cerebellum and anterior horns of the medulla it has been proposed that specific glial cells (e.g., Bergmann glia in the cerebellum) may function as catalysts for the capture and deposition of hemosiderin [2, 3]. Neuropathological examination has showed neuronal loss with scaring, reactive gliosis, ovoid-shaped bodies, demyelination and meningeal thickening [3]. At intracellular level, mitochondria seem to be the most affected organelles [4].

Classically, SS develops after the fourth decade of live carrying progressive bilateral sensorineural hearing loss (mainly high-frequency tones), cerebellar dysarthria, and ataxia (often affecting limbs and gait). Frequently associated signs are anosmia, pyramidal features, myelopathy, urinary dysfunction, frontotemporal dementia, and various cranial nerve palsies [5, 6].

First described by Hamill in 1908, SS remained a postmortem diagnosis, based in the brownish discoloration of the brain and leptomeninges [7]. The advances in neuroimaging, particularly the wide use of MRI, turned SS into an *in vivo* diagnosis and greatly increased the scientific interest in this condition. 

Analyzing the 270 cases registered until mid-2006 [8], the identified causes of SS were CNS tumors (21%), head and back trauma (13%), arteriovenous malformations/aneurisms (9%), previous neurosurgery (7%), brachial plexus injuries (6%), amyloid angiopathy (3%), and chronic subdural haematoma. In over a third of the cases, in spite of exhaustive investigation, it was not possible to establish a cause. 

The association between SS and oral anticoagulation treatment (OAT) is unclear and probably underreported. We present two clinical cases depicting the disparities in clinical presentation and progression of SS in patients under OAT.

## 2. Case Presentation

### 2.1. Patient 1

A 74-year-old woman presented with complaints of slowly progressive imbalance, bilateral hearing loss, anosmia, and occasional dysphagia. Although the patient suffered from these conditions for at least 8 years, she described a faster worsening in the last 6 months. The patient had received a mechanic mitral valve due to severe stenosis 30 years before and was under OAT ever since. At the age of 64, she fell twice and sustained minor head trauma in both occasions. Two years prior to observation she had a minor lumbar trauma and CT scans were not performed at the time.

The neurological examination revealed mild dysarthria, anosmia, severe bilateral sensorineural hearing loss, ataxia in both upper and lower limbs, and slow, wide-based walk with the need for external support. The rest of the examination was unremarkable.

**Figure 1:**
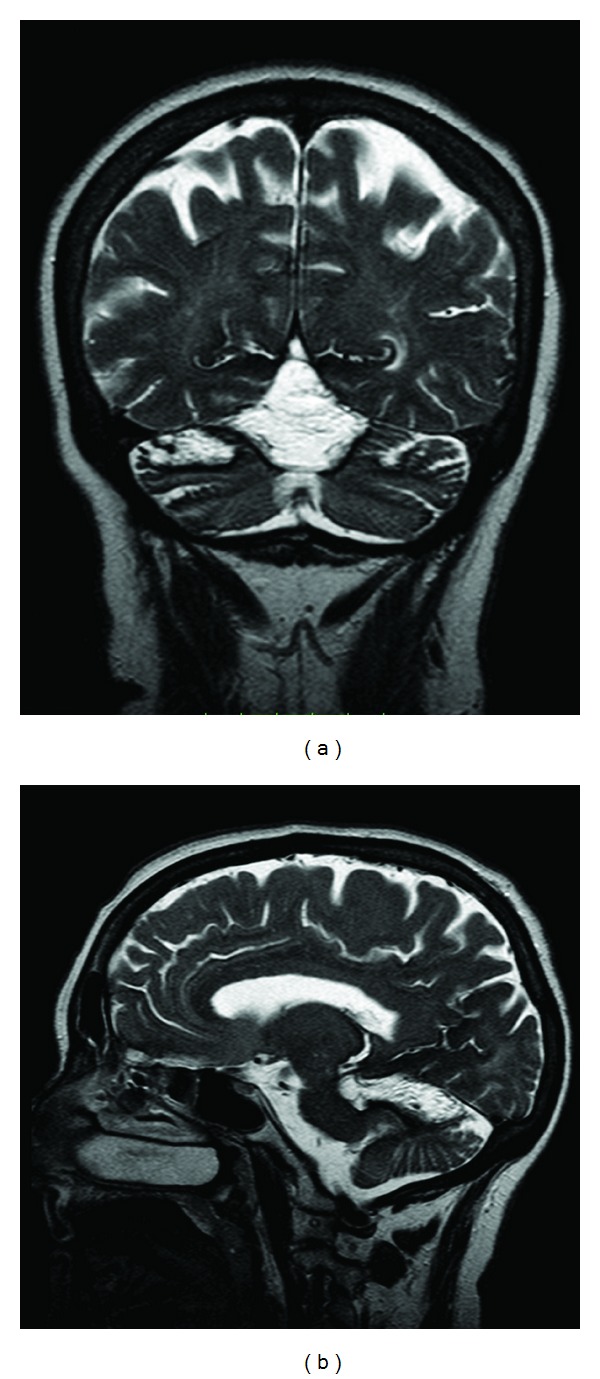
The coronal (a) and parasagittal (b) T2-ponderation MRI from patient 1 show a large clastic lesion over the vermis, marked cerebellar atrophy, and iron-related hypointensities around cerebellar hemispheres and brainstem.

**Figure 2:**
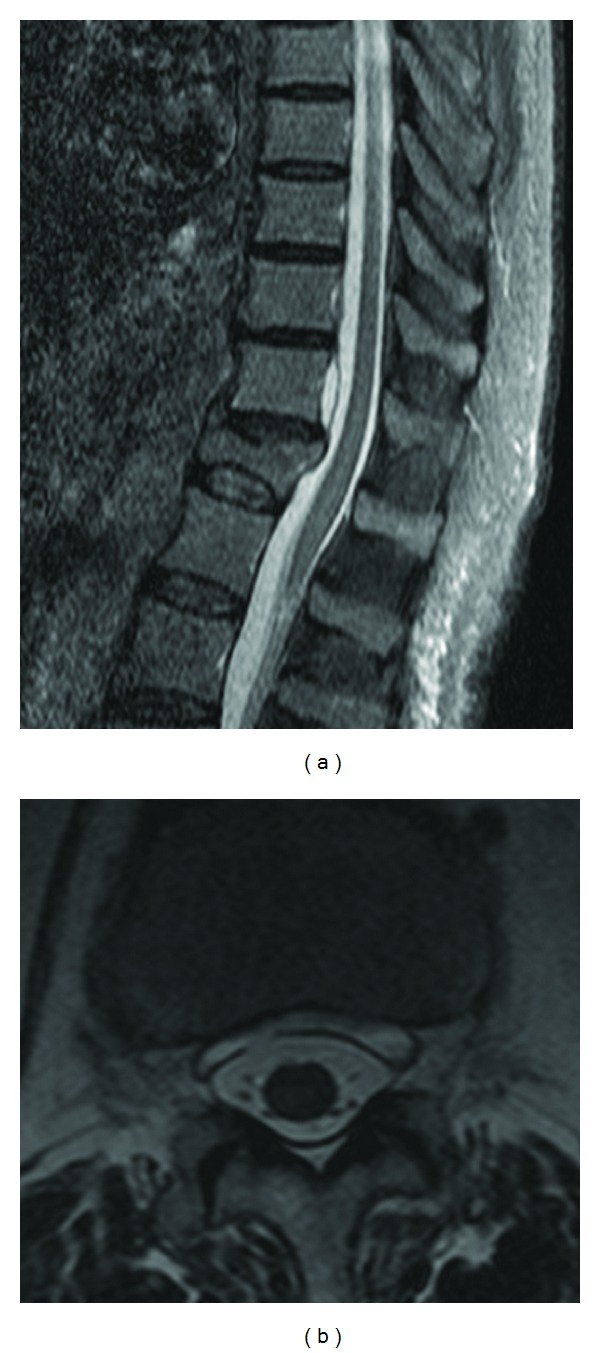
A sagittal STIR MRI (a) reveals an anterior subdural haematoma at T11 level with adjacent fracture and collapse of the 12th thoracic vertebral body, probably due to trauma. In this T2-pondered axial slice (b) there seems to be continuity between the haematoma and the subarachnoid space.

Brain MRI showed a large clastic lesion over the vermis, marked atrophy of the entire cerebellum, and hemosiderin around the cerebellar cortex, brainstem, and vestibulocochlear nerves (Figure 1). The spinal MRI disclosed a collapse of the T12 vertebral body and a chronic subdural haematoma at the level of T11 (Figure 2). Cerebral angiography failed to show any sources of bleeding or vascular malformations. Lumbar puncture (LP) could not be attempted due to high bleeding risk.

With the unequivocal diagnosis of superficial siderosis and after discussing the therapeutic options with the patient and relatives, we chose not to pursue any invasive procedures or followup exams. Unfortunately, the patient has become unable to walk due to ataxia and deafness progression.

### 2.2. Patient 2

A 72-year-old woman was referred due to behavioural changes, unsteadiness, and frequent falls over the previous year. She had initiated OAT two years prior to referral after the diagnosis of paroxysmal atrial fibrillation.

According to relatives, over the previous year the patient had become more listless, neglecting work and domestic activities. She also developed hyperreligious behaviour, anxiety, loss of appetite, complaints of mild lower limb weakness, and falls. Later, she started to suffer periods of agitation, confusion, and visual hallucinations and was started on antipsychotic drugs. Progressively, she lost the ability to walk and became confined to a wheelchair. 

At the time of admission, she presented disorientation, apathy, apraxia, short-term memory deficit (MMSE = 7), anosmia, and mild flaccid proximal paraparesis and muscular atrophy. Patellar reflexes and plantar responses were normal. Urinary incontinence was noted. There were no signs of sensitive impairment. 

**Figure 3:**
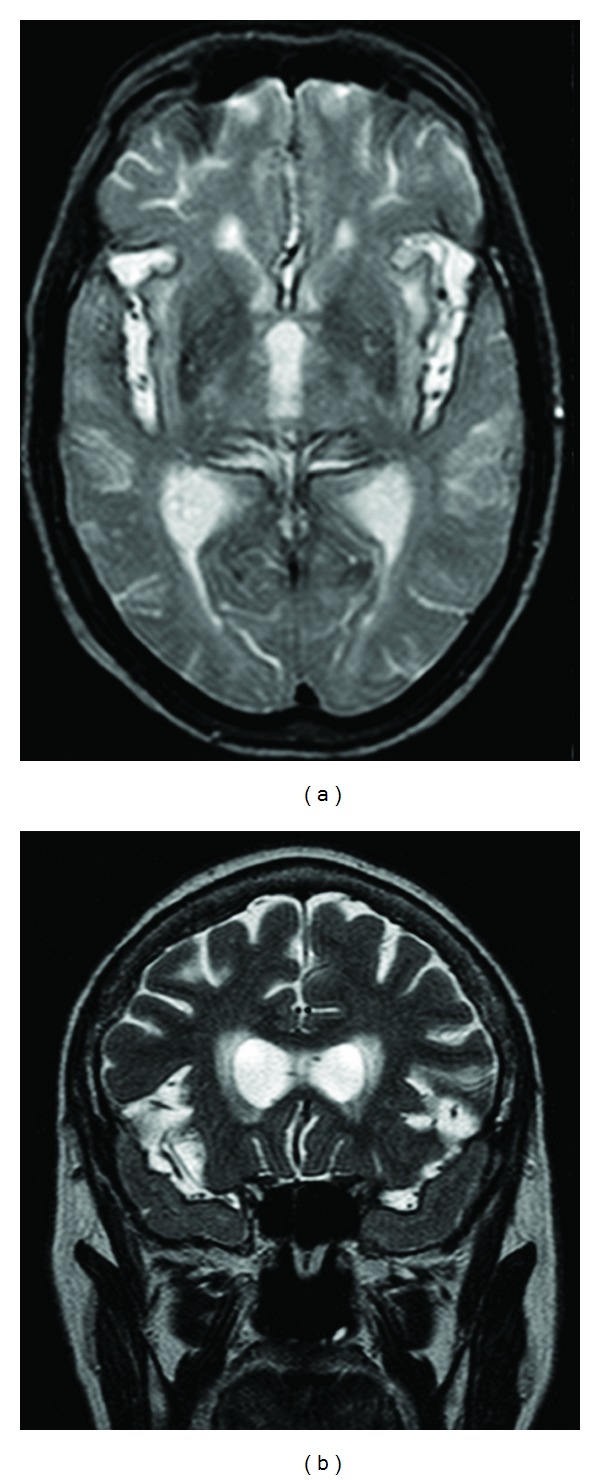
T2 sequences in axial (a) and coronal (b) slices illustrate the widespread of hypointensities in the bilateral frontotemporal cortex of Patient 2, thus suggesting strong iron deposition.

A general workup including vitamin levels, thyroid function, and tumour markers was normal. A 24-hour Holter monitor test showed no evidence of paroxysmal atrial fibrillation. EEG showed patterns of diffuse cerebral dysfunction with no epileptic activity. A full-body CT scan and a breast ultrasound were unremarkable. A brain MRI revealed T2*-pondered hypointensities around cerebral cortex (with a bilateral frontotemporal and insular predominance) and around the brainstem suggesting deposition of iron-containing products (Figure 3). An angioMRI did not reveal any source of bleeding.

**Figure 4:**
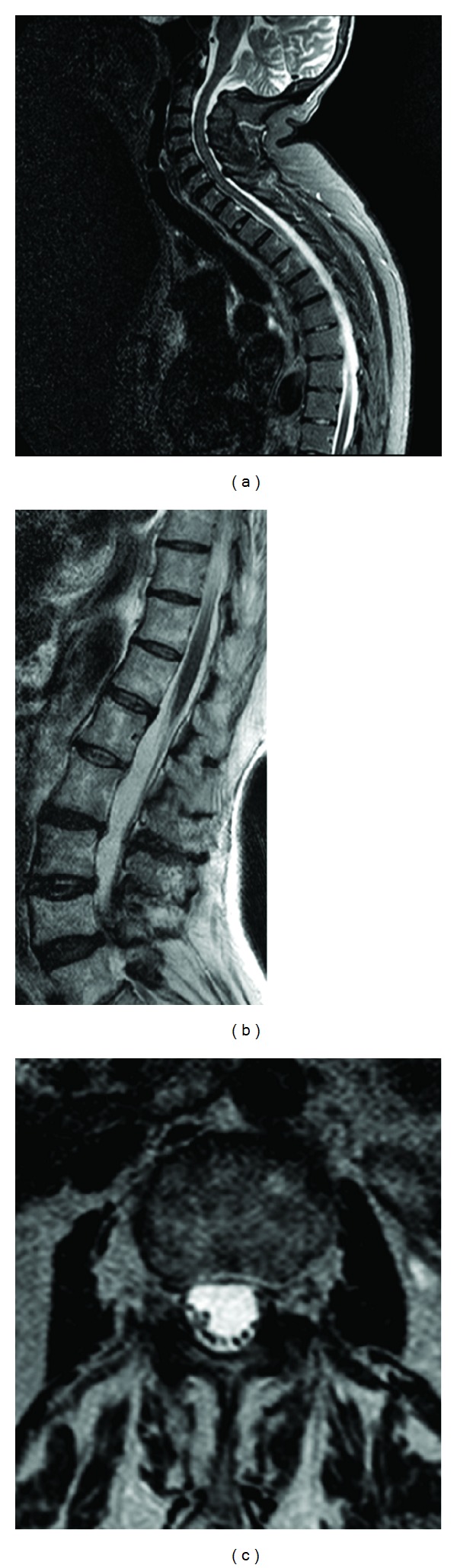
Hypointensities around the brainstem and all along the spinal cord are depicted in both sagittal MRI STIR-adquired images ((a) and (b)). The thecal *cul-de-sac* is occupied by iron-containing debris (b). There are also signs of arachnoiditis such as lumbar nerve root clumping ((b) and (c)).

Spinal MRI showed T2* pondered hypointensities all along the spinal cord with thecal iron accumulation between L4 and S1. There were also signs of arachnoiditis between L2 and L4 (Figure 4).

OAT was suspended and CSF analysis revealed xanthochromia, 4500 red blood cells (RBC)/*μ*L, 61 mg/dL in proteic concentration, and normal glucose level. CSF VDRL was negative and there were no neoplastic cells.

Considering all data, SS became the most likely diagnosis. As no source of bleeding could be found and due to the high risk of falls, we chose not to resume OAT at hospital discharge and she was medicated with fluoxetine and rivastigmine.

One month after discharge she had clear cognitive improvement and regained the ability to walk with assistance for short distances. Bilateral fasciculations and atrophy of thigh muscles, patellar hyperreflexia, and mild spasticity with flexor plantar responses were found. A new LP was performed, with no RBC in CSF and normal levels of glucose and proteins. Measurement of iron and ferritin in CSF could not be performed.

Two months after discharge, the patient walked with the help of a cane and had resumed domestic activities. Hypoacusis, not noticeable at hospital discharge, became a chief complaint. She still presented occasional fasciculations in thigh muscles. The patient underwent a second neuropsychological evaluation in which MMSE was again performed scoring a total of 26. An upper and lower limb EMG was within normal ranges, with no signs of fasciculations.

A followup brain and spinal MRI performed nine months after discharge did not show significant changes in the volume and distribution of iron byproducts. Six months since discharge and over the eighteen months of followup, the patient continued to carry an almost normal and independent live, although maintaining complaints of bilateral hypoacusis and anosmia.

## 3. Discussion

Patient 1 depicts the most recognizable presentation of SS: slowly progressive cerebellar ataxia and hypoacusis in a patient with a history of head trauma and compatible neuroimaging. After lumbar trauma, with the formation of a chronic subdural haematoma SS progressed more rapidly probably due to increased bleeding and chronic iron overload in the CSF. Prolonged use of OAT is likely to have contributed as a prohaemorrhagic risk factor. The strong deposition of hemosiderin along vestibulocochlear nerves and the cerebellum documented by imaging justifies the clinical presentation. In this case, no source of bleeding could be disclosed and the natural course of the disease could not be altered. Finsterer et al. [9] portrayed a similar patient where the risks of OAT cessation outweighed the possible benefits.

In contrast, patient 2 presented a fairly singular form of SS. The disease manifested itself as a rapidly progressive dementia combined with proximal paraparesis, initially flaccid with atrophy and fasciculations, then spastic. Preferential deposition over frontal, temporal, and insular cortices, lumbar and sacral spinal segments, and adjacent arachnoid seems to draw a parallel with the clinical symptoms. Isolated cognitive deficits have been reported in two patients suffering from SS [10]; however overt dementia is still an exceptional form of presentation. Paraparesis may be due to iron-related injury in lumbar anterior horns and peripheral nerves, with dysfunction of upper and lower corticospinal neurons leading to atrophy and fasciculations. These features were already observed and described in SS patients [11].

Although it was not possible to plainly establish the starting point or bleeding source, OAT seemed to play a crucial role in both clinical cases. In the first case it may be an aggravating factor together with trauma, facilitating the formation of bleeding sites from dural tearing or blood-filled cavities near the CNS. Likewise, patient 2 presented with no risk factor for SS or haemorrhage other than OAT and its withdrawal led to substantial clinical improvement. It is possible that by reducing the amount of blood entering subarachnoid space clearance of iron byproducts and neuronal plasticity ensued, therefore reducing symptom severity to a great extent.

Two cases of SS [12, 13] in patients under OAT where no haemorrhage source was found were described. In both cases the course of the illness was, at least partially, hindered by the suspension of OAT. However, we are not aware of any case where suspension of OAT led to such a clear clinical improvement as observed in Patient 2.

Generally considered a rare disease, several clinical cases with considerable clinical variance have been reported worldwide. In a population-based cohort of nondemented individuals between 65 and 75 years old the prevalence of SS was found to be 0.7%, and it was closely associated with MRI signs of cerebral amyloid angiopathy [14]. As the use of MRI becomes widespread in modern medicine it is to be expected that the finding of SS (and asymptomatic patients) will increase.

In theory, all the subpial CNS locations (and some not subpial structures, such as anterior horn cells) may be affected and different symptoms have been linked to SS. Epilepsy [15], psychosis [16], optic and trigeminal neuropathy, nystagmus, headache, radiculopathy, tethered cord syndrome caused by spinal venous hypertension, and pain related to arachnoiditis [6] were all associated with SS. Patients with SS presenting as a phenocopy of amyotrophic lateral sclerosis [17] or adult-onset spinocerebellar ataxia [18] have been depicted and certain authors proposed that SS should be excluded in any doubtful case. 

It is noteworthy that no correlation can be made between time of progression and the appearance of symptoms and an asymptomatic stage may last for decades [19]. In the same manner, the clinical severity of SS is not directly related with the amount of iron-magnetic substances found through neuroimaging studies [20].

The benchmark treatment of SS is surgical closure of the haemorrhagic source [21], which frequently, but not always [17], arrests the disease progression. Nonetheless, sporadic partial recoveries are described. The use of iron chelators has been attempted, with deferiprone [22] being the most promising drug. Unfortunately, studies are inconclusive and more trials are needed.

In summary, albeit SS classical features are slowly progressive sensorineural deafness, cerebellar ataxia, and pyramidal involvement, the form of presentation and its progression are considerably variable and include rapidly progressive dementia, psychosis, or lower motor neuron signs. Increased use of MRI as diagnostic weapon has put in evidence that SS may be a rare diagnosis but a more common disease than previously thought. The mainstay of treatment is the localization and surgical correction of the bleeding source. However, factors such as oral anticoagulation therapy can act as contributing and may even be causative agents. Therefore, withdrawal of oral anticoagulants or optimization of INR, whenever possible, may be considered as disease-modifying measures.

## Abbreviations

CNS: Central nervous system
CSF: Cerebrospinal fluid
CT: Computerized tomography
EEG: Electroencephalography
EMG: Electromyography
INR: International normalized ratio
LP: Lumbar puncture
MMSE: Mini-Mental State Examination (Folstein)
MRI: Magnetic resonance imaging
OAT: Oral anticoagulation therapy
RBC: Red blood cells
SS: Superficial siderosis.
